# Glycerin in Bromidrosis

**Published:** 1915-06

**Authors:** 


**Glycerin in Bromidrosis.** Benians, Lancet, Dec. 5, 1914. After failure of cleanliness and various powders, this was efficient. He thinks it deters bacteria from forming indol, etc., locally and by furnishing an acid medium (What acid?) instead of an alkaline, for the skin of the feet (Is perspiration alkaline?)

